# Can mammographic features predict invasive carcinoma at screening assessment?

**DOI:** 10.1186/bcr2982

**Published:** 2011-11-04

**Authors:** ND Forester, P Burrows, A-M Wason

**Affiliations:** 1 Pennine Breast Unit, St Luke's Hospital, Bradford, UK

## Introduction

In our institution, 30% of screen-detected DCIS diagnosed by 14G core biopsy will be upgraded to invasive carcinoma at definitive surgery, necessitating subsequent sentinel node procedure to complete staging. This could be decreased by first-line large-bore vacuum-assisted biopsy. Prior to introducing this, we reviewed patient factors, aiming to identify subsets of patients who may benefit from first-line vacuum-assisted biopsy.

## Methods

A retrospective review of B5a diagnoses at initial biopsy, April 2009 to March 2010. Univariate and multivariate logistic regression analysis was performed to analyse the impact of patient, radiographic and histological factors on the B5b upgrade rate.

## Results

A total of 51 patients had DCIS diagnosed on initial core biopsy. Fifteen were upgraded to invasive carcinoma following surgical excision. No association between patient age, screening round, grade of DCIS, calcification pattern (focal vs. diffuse) and ER/PR status was found. Mammographic calcification size did significantly predict upgrade in univariate analysis (OR = 1.04 per mm increase in size of calcification; 95% CI = 1.01 to 1.07; *P *= 0.02) and in multivariate analysis when adjusting for patient age and calcification pattern (OR = 1.04; 95% CI = 1.00 to 1.07; *P *= 0.043). Stratification of mammographic calcifications showed that clusters >20 mm in size have 16.6 times the odds of upgrade compared with calcifications <10 mm (95% CI = 1.38 to 200.02; *P *= 0.027).

## Conclusion

Size of calcifications on mammography is a significant predictor of upgrade to invasive carcinoma. Increases in calcification of 10 mm increase the odds of upgrade to invasive carcinoma by 34%. Given the significantly increased upgrade rate for calcifications over 20 mm, first-line vacuum-assisted biopsy in this subset of patients at initial screening assessment may prove cost-effective.

